# Multi-Frame Based Homography Estimation for Video Stitching in Static Camera Environments

**DOI:** 10.3390/s20010092

**Published:** 2019-12-22

**Authors:** Keon-woo Park, Yoo-Jeong Shim, Myeong-jin Lee, Heejune Ahn

**Affiliations:** 1 The Information Technology & Mobile Communications Biz., Samsung Electronics, Suwon-si, Gyeonggi-do 16677, Korea; kw92.park@samsung.com; 2 School of Electronics and Information Engineering, Korea Aerospace University, Goyang-si, Gyeonggi-do 10540, Korea; yoojeong2018@kau.kr; 3 Dept of Electrical and Information Engineering, Seoul National University of Science and Technology, Seoul 01811, Korea; heejune@seoultech.ac.kr

**Keywords:** homography estimation, video stitching, multi-frame based homography, representative feature point, video alignment

## Abstract

In this paper, a multi-frame based homography estimation method is proposed for video stitching in static camera environments. A homography that is robust against spatio-temporally induced noise can be estimated by intervals, using feature points extracted during a predetermined time interval. The feature point with the largest blob response in each quantized location bin, a representative feature point, is used for matching a pair of video sequences. After matching representative feature points from each camera, the homography for the interval is estimated by random sample consensus (RANSAC) on the matched representative feature points, with their chances of being sampled proportional to their numbers of occurrences in the interval. The performance of the proposed method is compared with that of the per-frame method by investigating alignment distortion and stitching scores for daytime and noisy video sequence pairs. It is shown that alignment distortion in overlapping regions is reduced and the stitching score is improved by the proposed method. The proposed method can be used for panoramic video stitching with static video cameras and for panoramic image stitching with less alignment distortion.

## 1. Introduction

With the development of information and communications technology (ICT), there is a growing demand in various fields, such as virtual reality (VR), image security, entertainment, aerial image, and mobile applications for panoramic images and videos with a wide angle of view [1,2,3,4]. Algorithms for aligning and stitching images into a seamless mosaic image are widely used in computer vision [5,6,7,8]. The estimation of the correct alignment relating various pairs of images, the choice of a final compositing surface for warping aligned images, and the seamless cutting and blending of overlapping images are required for image stitching even in the presence of parallax, lens distortion, scene motion, and exposure differences.

Recently, as the demand for the VR application of 360-degree video increases, existing research on image stitching is being extended to video stitching [9,10,11]. However, due to the influences of illumination change and time-varying noise, it is difficult to accurately estimate a homography between video frames. The inaccurate estimation of a homography may cause alignment distortion and jitter over the entire stitched video sequence [12]. Furthermore, homography estimation by individual video frame may increase the computational complexity.

Conventional studies to improve the performance of video alignment for stitching can be classified according to the motion characteristics of cameras. Since homographies between cameras is fixed in static camera environments, the homography extracted from the first input frame pair can be used in all subsequent frames [13,14,15]. In dynamic camera environments, the homography between two cameras may change with time. Therefore, the homography between the first input frames of a pair of video sequences to be stitched is extracted, the motion for each video sequence is calculated using the optical flow algorithm, and the new frame-specific homography is updated using the initial homography and the frame-specific motion [16,17].

In all existing types of video stitching methods, a homography between the first frame pair is used for the subsequent frame pairs. In a static camera environment, an inaccurate initial homography can affect the stitching performance of the entire video sequence pair. In a dynamic camera environment, the homography of the following frame pair may be inaccurate because it is calculated using an inaccurate initial homography and an estimated camera motion. Thus, if the initial homography is inaccurate, the stitching performance of the entire video sequence pair can be degraded irrespective of the accuracy of the estimated camera motion. Therefore, in order to improve the video stitching performance, it is necessary to improve the accuracy of the matching between frame pairs, which is the basis of the initial homography estimation.

Finding correspondences across cameras has been attempted through pixel-by-pixel mapping between two images [18] and by using feature detection methods such as the scale invariant feature transform (SIFT) [19] or speeded-up robust features (SURF) [20]. The pixel-by-pixel matching technique is sensitive to radiation variation between images and has difficulties, due to uncertainties in camera parameters. Although feature-based matching is more robust against radiation variation, it may not work well where camera parameters, exposure, and illumination conditions are very different across cameras. Moreover, in both techniques, matching becomes ambiguous when the images have plain textures or few feature points.

Finding correspondence matching of multi-view video sequence pairs has been attempted [21,22,23,24,25,26,27,28]. The independent application of image matching methods to each pair of video frames has the same drawbacks of matching images [21]. The temporal information of moving objects has been applied to estimate homography [22,23,24,25,26,27,28]. The centroids [22,23,24] or motion trajectories [25] of moving objects in different cameras are used as feature points for homography estimation. However, these methods require solving object tracking and data association problems across camera and cannot be applied to video sequence pairs without moving objects. Another approach to matching between a video sequence pair is to use activity features of moving objects [26,27,28]. Activity features are defined at each pixel position as a temporal series of binary values indicating the existence of foreground objects. The corresponding pixels in a different view that has the most similar activity features are selected for matching. Although these methods are robust against arbitrary orientations and zoom levels of cameras and illumination conditions, they have the limitation that moving objects must exist in the overlapped regions in the video sequence pair.

To improve the quality of stitched video sequences, it is necessary to find correct alignment parameters between cameras while suppressing the wrong feature point extraction from time-varying noise. In this paper, a novel multi-frame based homography estimation method is proposed for correct video alignment in static camera environments. A homography that is robust against spatio-temporally induced noise can be estimated by intervals, using feature points extracted during a predetermined time interval. To find spatially consistent representative feature points for matching of a pair of video sequences, representative feature points with the largest blob response are selected among feature points extracted in a predetermined time interval for quantized location bins. To find temporally consistent feature points over the predetermined time interval, SURF feature locations are quantized into finite two-dimensional histogram bins for the accumulation of their numbers of occurrences. After matching representative feature points from each camera, a homography for the interval is estimated by random sample consensus (RANSAC) using feature points in the histogram bins, with their chances being sampled in proportion to their histogram counts.

For the evaluation of the accuracy of the estimated homography, the frames of the left camera are warped into the planes of the frames of the right camera using the estimated homography. The warped left frames and the right frames are average blended into stitched frames without a seam-line estimation. The alignment error of the video sequences stitched using the proposed method is compared with that of video sequences stitched using a per-frame method.

The contribution of the proposed method is three-fold. First, the proposed method can reduce alignment error due to inaccurate estimation of initial homography in stitched video sequences. Second, the proposed method can be effectively applied to video sequence pairs, of which confidence for extracted feature points is not sufficiently high, due to noise or the plain texture of the video sequences. Finally, the proposed method does not require moving objects for homography estimation, and can be used for general video sequence pairs with or without moving objects.

This paper is organized as follows: In Section 2, a multi-frame based homography estimation method is proposed for video stitching. In Section 3, the performance and effectiveness of the proposed homography estimation method are evaluated. Finally, in Section 4, conclusions and future work are presented.

## 2. Multi-Frame Based Homography Estimation

**Figure 1:**
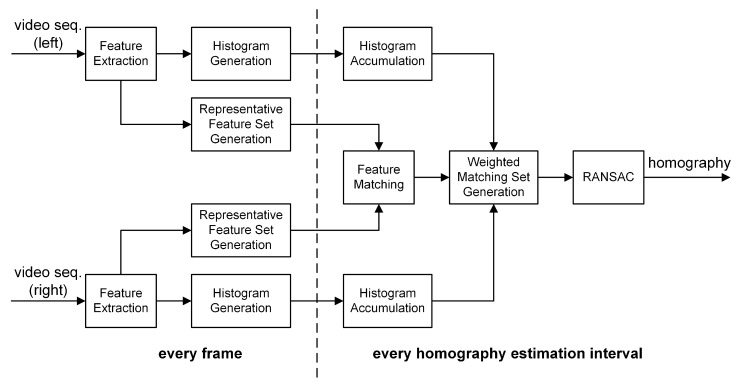
Proposed multi-frame based homography estimation algorithm.

In this section, a novel multi-frame based homography estimation algorithm, shown in Figure 1, is proposed for stitching a pair of video sequences captured by static cameras. Feature points are extracted from a pair of video sequences to stitch and a matching process is applied to representative feature points with the largest blob response in quantized location bins. A histogram of two-dimensional quantized locations of feature points is calculated over a predetermined time interval, the homography estimation interval $T_{e}$, for each video sequence to be stitched. Finally, for homography estimation, RANSAC algorithm is applied to the matched representative feature points, with their chances of being sampled proportional to their histogram counts.

### 2.1. Selection of Representative Feature Points

**Figure 2:**
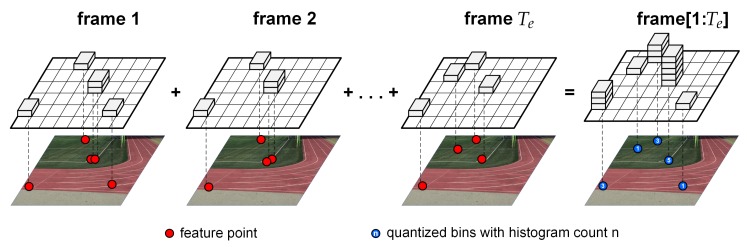
Feature location histogram generation for multi-frame interval.

In the first step, as shown in Figure 2, feature points are extracted using the SURF algorithm from video frames over the homography estimation interval $T_{e}$ for each video sequence. The SURF algorithm interpolates a scale-space extremum’s location and scale to sub-pixel accuracy to form an image feature. To find spatially consistent feature points to match a pair of video sequences, representative feature points are selected among feature points extracted in the homography estimation interval. The locations of feature points are quantized into location bins. The feature point with the largest blob response in each location bin is selected as the representative feature point for the bin during the interval.

A square quantized location bin $G_{v}^{r}$, of which the upper-left is at location v, is defined for the selection of a representative feature point, of which the upper-left is at v. For spatially consistent feature point selection, the bin sets the unit area where neighboring feature points are treated as a group. The width $\Delta_{r}$ of the bin should be determined such that neighboring feature points are of similar signal characteristics and the number of feature points in the quantized location bin in the homography estimation interval is sufficient for selection. In our study, the width $\Delta_{r}$ is set to one pixel.

For a quantized location bin $G_{v}^{r}$, if there exists more than one feature point in the location bin, a feature point with the largest blob response is selected as the representative feature point for the location bin in the homography estimation interval, as follows:

(1) $$p_{v}=\underset{l\left(p\right)\in G_{v}^{r},p\in S_{n}}{arg max}R\left(p\right),$$

where l(p) and R(p) represent the location and the blob response function of a feature point p, respectively. The blob response is the approximated determinant of the Hessian in SURF [20]. $S_{n}$ represents the set of feature points in the nth homography estimation interval $T_{n}$.

Representative feature point selection is independently performed on each sequence of a video sequence pair in the homography estimation interval. These representative feature points are spatially consistent during the interval and are used for matching without the other feature points, which makes the matched feature points more reliable and the matching faster.

### 2.2. Interval-Based Histogram Generation for Feature Points

In the second step, to find temporally consistent feature points, a histogram of SURF feature locations is generated. SURF feature locations are quantized into two-dimensional histogram bins, and the number of occurrences of feature points in each histogram bin is counted over the time interval. A square quantized histogram bin $G_{v}^{h}$, with width $\Delta_{h}$, is defined for the generation of a histogram of feature locations, of which the upper-left is at v.

A set of feature points in the histogram bin $G_{v}^{h}$ in the homography estimation interval $T_{n}$ is a subset of $S_{n}$ and is defined as follows:

(2) $$S_{n,v}=\left\{p|l\left(p\right)\in G_{v}^{h},\forall p\in S_{n}\right\}.$$

In the interval $T_{n}$, the histogram count for the quantized histogram bin $G_{v}^{h}$ where a feature point p is located in, is calculated as follows:

(3) $$h^{n}\left(p\right)=\left|S_{n,v}\right|,$$

where |·| represents the cardinality of a set.

The histogram generation is independently performed on each sequence of a video sequence pair in the homography estimation interval. The width of the histogram bin is set to a half pixel, i.e., $\Delta_{h}=0.5$. Although, in our study, the resolution of quantized location bins for representative feature point selection is lower than that for histogram generation, the quantized histogram bins for histogram generation are integer-quantized for simplicity of illustration, as shown in Figure 2.

### 2.3. Matching Representative Feature Points

**Figure 3:**
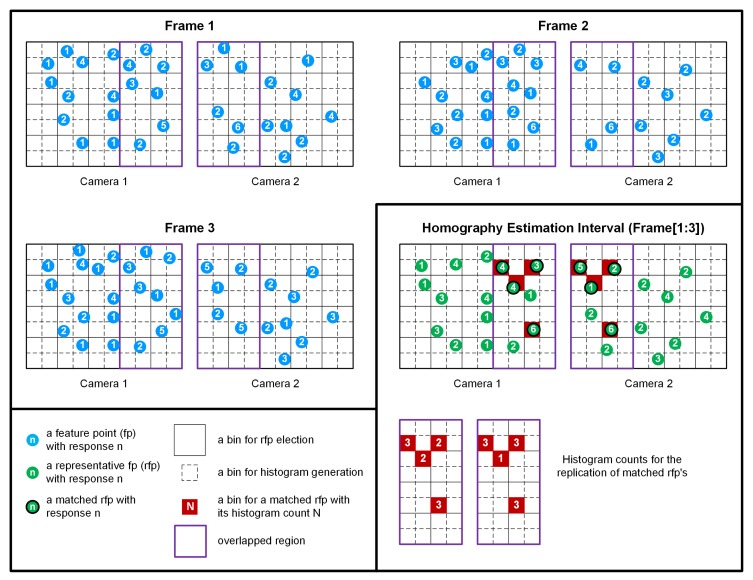
Interval-based matching of representative feature points and their histogram generation.

In the third step, nearest neighbor matching is performed on the representative feature points from video sequence pairs in the homography estimation interval [19,20,29], which results in a set of matched representative feature point pairs, as shown in Figure 3. The matching strategy performs thresholding on the distance ratio between the first and the second nearest neighbors. The distance ratio threshold of 0.3 was used for our experiments. Because per-frame feature matching may not result in good matching pairs for homography estimation in static camera environments, due to the dynamic change of noise and illumination, it is not performed in our study.

The sub-figures titled Frames 1, 2, and 3 in Figure 3 illustrate the weakness of per-frame feature matching. Because there may not exist enough feature point pairs for the homography estimation in the overlapped area, especially for video sequences with plain texture, it may be difficult to differentiate consistent and strong feature points from others. However, in the right-bottom sub-figure of Figure 3, Frame [1:3], because the proposed algorithm accumulates feature points over the homography estimation interval, there exist enough feature points in the overlapped region for matching. Furthermore, from the interval-based matching results, the matched representative feature points may be easily differentiated from each other in terms of their temporal consistency by using their histogram counts. The quantized resolutions of feature locations are used only for representative feature point selection and histogram generation. The feature points used for matching and RANSAC have their original resolutions with no quantization.

### 2.4. Homography Estimation by Histogram Weighted RANSAC

In the final step, the homography *H* between a pair of video sequences is estimated based on the simple outlier elimination technique, RANSAC [30], with the matched representative feature point pairs. Although RANSAC is largely insensitive to outliers, it will fail if the fraction of outliers is high. It is desirable for temporally consistent matched representative feature point pairs, which are robust against noise or have larger blob response, to contribute more to the homography estimation during RANSAC iteration. Thus, each matched representative feature point pair is replicated multiple times into a dataset for RANSAC by considering their histogram counts and blob responses and their averages for all the matched representative feature points.

The average blob response for all the feature points is calculated asfollows:

(4) $$r_{avg}=\frac{1}{2M}\sum_{i=1}^{|S_{n}^{m}|}\left\{R\left(p_{i}^{l}\right)+R\left(p_{i}^{r}\right)\right\},$$

where $p_{i}^{l}$, $p_{i}^{r}$, and *M* represent the ith left and right matched representative feature points and the number of pairs of matched representative feature points, respectively.

The average histogram count for all the matched representative feature points is calculated as follows:

(5) $$c_{avg}=\frac{1}{2M}\sum_{i=1}^{|S_{n}^{m}|}\left\{h^{n}\left(p_{i}^{l}\right)+h^{n}\left(p_{i}^{r}\right)\right\}.$$

The replication number of the ith matched representative feature point is calculated as follows.

(6) $$n_{i}=\frac{R\left(p_{i}^{l}\right)\cdot h^{n}\left(p_{i}^{l}\right)+R\left(p_{i}^{r}\right)\cdot h^{n}\left(p_{i}^{r}\right)}{2\cdot r_{avg}\cdot c_{avg}}.$$

For homography estimation using the proposed histogram weighted RANSAC algorithm, four feature correspondences are selected from the histogram weighted RANSAC dataset. The homography H between them is calculated using the direct linear transformation (DLT) [31]. This process is repeated with n=1000 trials and the candidate with the maximum number of inliers whose projections are consistent with H within a tolerance of three pixels is updated every trial.

## 3. Experimental Results

In order to evaluate the performance of the proposed multi-frame based homography estimation method, the video alignment results of the proposed method are compared with those of the per-frame stitching method [6,20]. The proposed method can improve the accuracy of homography by matching representative feature points of the largest blob responses and by replicating these matches for RANSAC according to their histogram counts and strengths of blob responses. Alignment distortion is compared in the overlapped region in the stitched video frames, which are aligned using the estimated homography without post-processing of seam-line selection, gain compensation, or multi-band blending used in [6,13,15]. The reason why the post-processing algorithms are not used is to leave unwanted alignment distortion from inaccurate homography unhidden.

Experiments for performance evaluation consist of three parts. The first experiment compares the alignment distortion and stitching score [17] of the aligned frames after applying the proposed and the per-frame method to daytime video sequences. The second experiment also compares the alignment distortion and the stitching score for noisy video sequences to test the robustness of the estimated homography against noise. The last experiment evaluates the influence of the homography estimation interval on the stitching score and the processing speed. In the first and second experiments, the homography estimation interval was set to 20 frames. The proposed and the per-frame methods were implemented using OpenCV3.2 library without GPGPU acceleration, and the computational speeds were measured on a computer with i7-7770 @ 3.6GHz CPU with 16 GB memory.

To evaluate the performance of the proposed algorithm, four daytime and two nighttime video sequence pairs, listed in Table 1, were used for the experiments. Each video sequence pair used in the experiments consists of two video sequences with overlapped regions taken by two static cameras with rotation and translation from each other. The focal lengths [$f_{x}$, $f_{y}$] of the cameras are [1472.9, 1465.3] and [2117.9, 2101.3], respectively. The optical centers of the cameras are [945.3, 576.9] and [946.6, 504.9], respectively. Each video sequence pair is stitched into a video sequence of one wide angle of view using homographies estimated by the proposed and the per-frame methods. Four daytime and two nighttime video sequence pairs are captured for the experiments. All the sequence pairs are 10 s long and are captured with a resolution of 1920 × 1080 at a frame rate of 30 Hz.

### 3.1. Statistics of Matched Representative Feature Points

**Figure 4:**
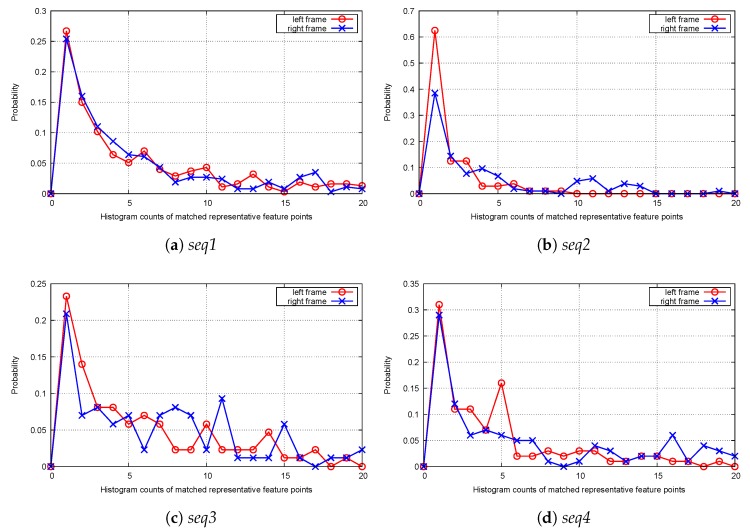
Probability distribution of histogramcounts ofmatched representative feature points (*T_e_* = 20).

After selecting representative feature points for the homography estimation interval, a matching experiment is performed using *seq1–4*. As shown in Figure 4, more than 20% of matched representative feature points occurred once during the homography estimation interval, while some matched instances occurred more than ten times. The matched points with small histogram counts can be considered as weak cases, while the others are stronger cases. From the interval-based matching results, it can be argued that the matched representative feature points can be easily differentiated from each other in terms of their temporal consistency by using their histogram counts.

The matches were visually inspected for their correctness in descending order of their reprojection distances. Most of the matches with reprojection distances exceeding five pixels are considered as incorrect in our experiments. The ratios of incorrect matches with the proposed method are 5.9%, 9.6%, 2.3%, and 6.0% for *seq1–4*, respectively. The worst ratios of incorrect matches in the per-frame method are 8.0%, 14.5%, 11.3%, and 10.2% for *seq1–4* during the homography estimation interval, respectively. Because the proposed method uses representative feature points consistent in the spatio-temporal direction for matching, the ratios of incorrect matches are lower than those in the per-frame method.

### 3.2. Performance Comparison for Daytime Video Sequence Pairs

The first experiment estimates homographies using the proposed and the per-frame methods for daytime video sequences and compares the alignment distortion and the stitching score for the video frames stitched by these methods.

The proposed method estimates the homography by the homography estimation interval; all the frames in the interval are aligned with the same homography. The per-frame method aligns each frame pair using the homography estimated for the pair.

**Figure 5:**
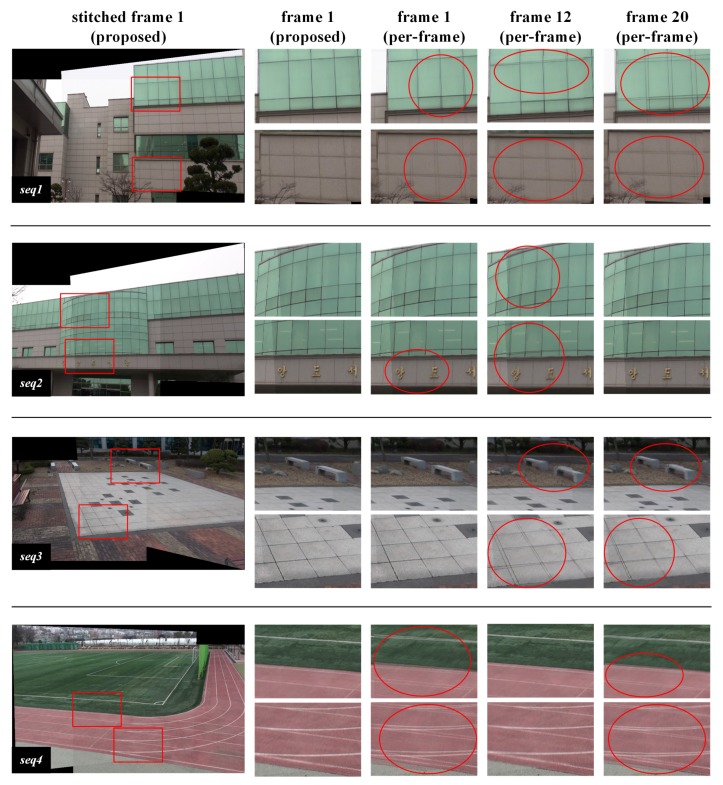
Stitching results for daytime video sequence pairs.

Figure 5 shows video frames stitched by the proposed and the per-frame methods in the first homography estimation interval. Because there is little change in the video signal in the temporal direction, a representative stitched frame for the interval is presented as a result of stitching by the proposed method. In this figure, the first frame in the homography estimation interval is selected as the representative stitched frame for the interval. In the per-frame method, since the homography used for stitching may vary from one frame to another, frames 1, 12, and 20 are selected for the presentation of stitching results in the interval.

Alignment distortion can be visually assessed when a frame warped by homography is superimposed on the other frame in the region where the frame pairs overlap. The first column in Figure 5 shows representative frames stitched by the proposed method. The red rectangles in the frame are regions where alignment distortion due to inaccurate homography estimation stands out. Areas with alignment distortion, caused either by the proposed or the per-frame method, are enlarged and displayed from the second to fifth columns. Using the homography estimated by the proposed method, alignment distortion in the overlapping regions of the stitched frames was hardly noticeable. However, it can be seen that alignment distortion occurs for almost every frame in the overlap region stitched by the homography estimated by the per-frame method.

In the per-frame method, noise and illumination change over time, which may affect the feature extraction and matching processes. As a result, the estimated homography may change every frame, degrading the quality of the entire stitched video sequence with jitter. On the other hand, the proposed method shows unnoticeable alignment distortion in the frames in the homography estimation interval. This is because the homography is estimated by selecting from feature points existing in the interval matched representative feature points that are robust against noise or illumination variation. The proposed method effectively filters noise components distributed in spatio-temporal directions through two processing stages. In the first step, feature points of relatively low blob response, which may be temporally induced by noise, are removed by selecting one representative feature point having the highest response for each quantized location bin in video frames in the homography estimation interval. In the second step, feature points frequently extracted in the same locations in the homography estimation interval are differentiated from infrequent feature points using a two-dimensional histogram of feature point locations. In the RANSAC based homography estimation process, replicating both spatially and temporally consistent matched representative feature points increases their possibilities of being sampled for homography estimation.

For quantitative evaluation of the video alignment results, stitching scores for stitched video frames, shown in Figure 5, are calculated and shown in Table 2. Since the stitching score represents the reprojection distance of matching feature points [13,16], the smaller the value, the higher the degree of matching. Given the use of multi-frame based matching by the proposed method, the set of matched feature points used for calculating the stitching score differs from that of the per-frame method.The per-frame method generates stitched frames with noticeable alignment distortion and high stitching scores. On the other hand, the proposed method generates stitched frames with unnoticeable alignment distortion and low stitching scores. The stitching scores of the proposed method are lower than those of the per-frame method also for the frames with unnoticeable alignment distortion in the per-frame method. As a result, the proposed method shows better alignment than the per-frame method for most stitched frames. Furthermore, the average stitching scores of the proposed method for the homography estimation interval are superior to those of the per-frame method. These results indicate that the interval-based homography estimated by the proposed method is more accurate than the frame-based homography estimated by the per-frame method.

**Figure 6:**
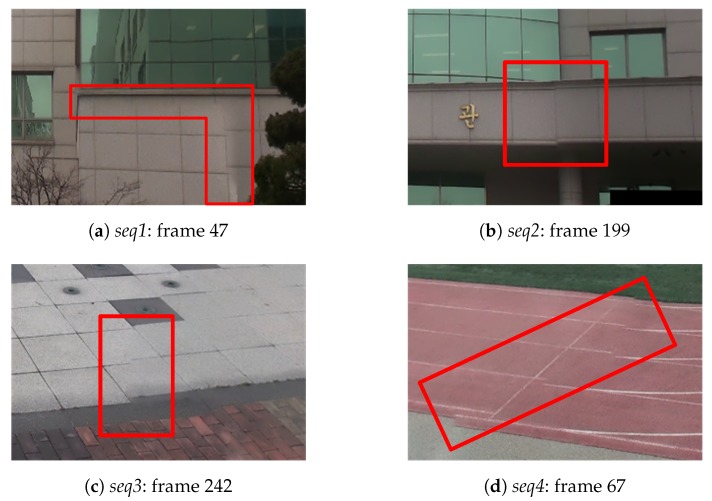
Misalignment distortion around seams by the per-frame method with seam estimation and advanced blending.

We used average blending on the overlapped region to present misalignment in this region due to an incorrect homography estimation. The seam selection and blending algorithms of advanced video stitchers can efficiently maintain the spatial and temporal consistency and remove any color discontinuities in overlapped regions [6,8,15,32]. However, these advanced algorithms cannot explicitly remove or minimize misalignments, though they can conceal or minimize discontinuities in the video signal. Nonetheless, these advanced algorithms still have discontinuities from misalignments in regions with periodic patterns or lines. Figure 6 shows cases of the use of the per-frame method where a discontinuity due to misalignment exists regardless of the use of the graph-cut based seam estimation [32] and multi-band blending [6].

### 3.3. Robustness Against Noise

**Figure 7:**
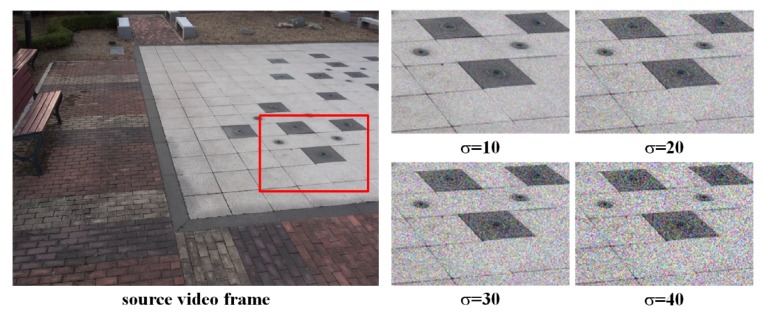
Examples of daytime video sequence with artificial Gaussian noise.

To evaluate the robustness against noise of the proposed method, the second experiment compares the alignment distortion and stitching scores of the proposed method with those of the per-frame method for daytime video sequences with Gaussian noise added and nighttime video sequences. As can be seen in Figure 7, noise-blended video sequences are created by adding noise with different variances to daytime video sequences. Homographies are estimated using the proposed and the per-frame methods for daytime sequences with noise and nighttime sequences, respectively. In order to evaluate the accuracy of the estimated homography, we assess the alignment distortion of frames stitched with the original video sequences using the homography estimated from noisy video sequences. Nighttime video sequences were captured in night vision mode of cameras; other experimental conditions were the same as those for daytime video sequences.

**Figure 8:**
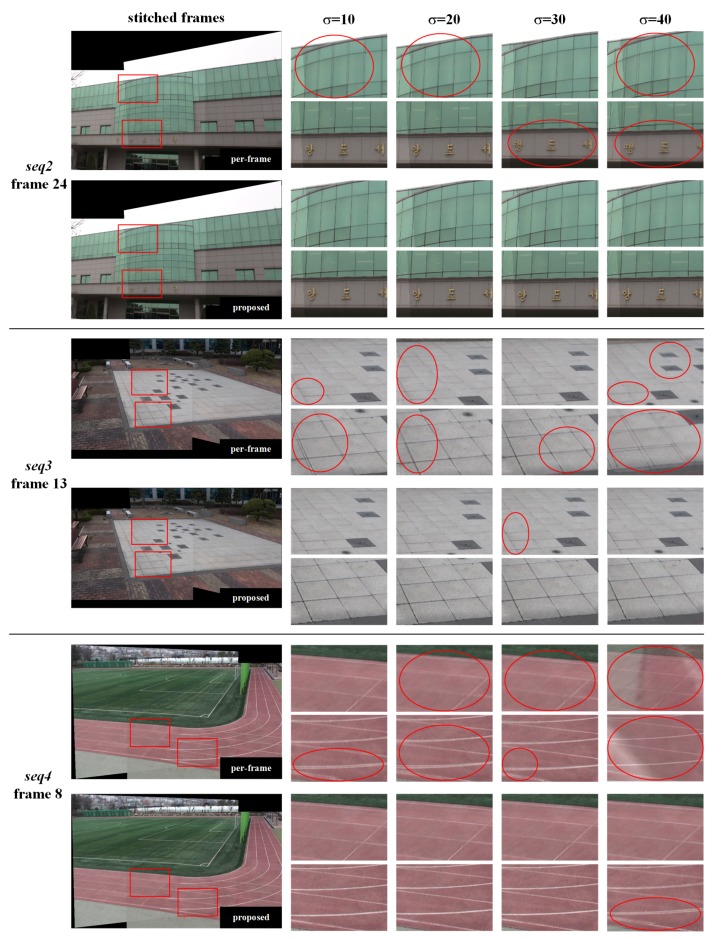
Stitching results for daytime video sequences with homographies estimated from video sequences with artificial noise.

Figure 8 shows stitching results with homographies estimated from noise-blended daytime video sequences, i.e., *seq2*, *seq3*, and *seq4*. As the variance of noise increases, the alignment distortion increases because the estimated homography becomes less accurate, due to the increment in the number of false feature points and the decrement in the number of real feature points. In the per-frame method, there is noticeable alignment distortion in all stitched video sequences. In the case of *seq4*, when the noise variance was 1600, the per-frame method resulted in a complete misalignment in the stitched frames due to a large error in the estimated homography. On the other hand, the proposed method has little or unnoticeable alignment distortion for all noise powers. This is because the proposed method performs matching and homography estimation using noise-resistant feature points of high blob responses and frequent occurrence in the homography estimation interval.

**Figure 9:**
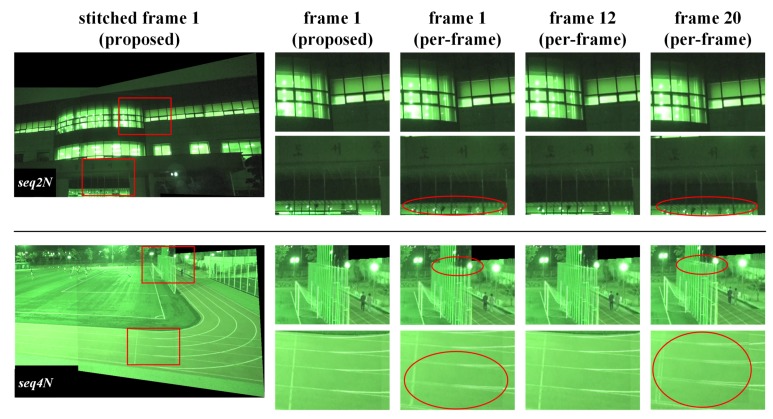
Stitching results for real nighttime video sequences.

For nighttime video sequence pairs, there may be noise amplification by night vision circuit or noise due to characteristics of illumination sources. In order to evaluate the influence of noise on homography estimation in the actual shooting environment, nighttime sequence pairs similar to *seq2* and *seq4* were captured and these pairs were stitched with the same process used for daytime sequence pairs. Figure 9 shows the stitching results for nighttime sequence pairs. In the per-frame method, alignment distortion due to inaccurate homography estimation still exists, but alignment distortion is unnoticeable in the frames stitched using the proposed homography estimation method. This means that the proposed method can accurately estimate homography even for nighttime sequence pairs with a lot of noise and can synthesize panoramic video frames with less alignment distortion.

### 3.4. Effect of Homography Estimation Interval on Stitching Performance and Processing Speed

**Figure 10:**
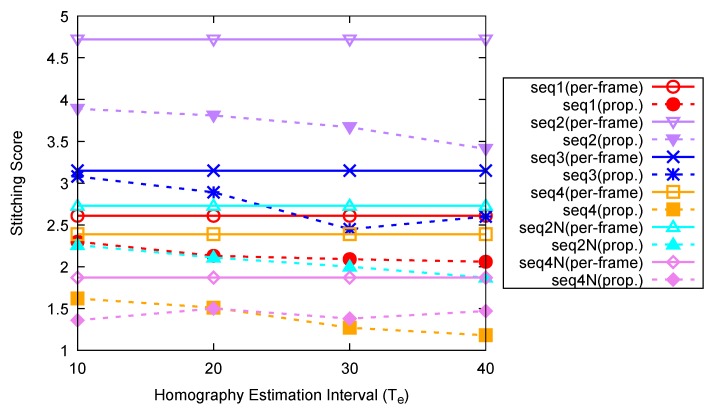
Average stitching scores for different homography estimation interval $T_{e}$.

In the third experiment, the effects of the homography estimation interval on the stitching score and the processing speed are evaluated. Homography estimation and stitching are performed with four different homography estimation intervals for *seq1–4*, *seq2N*, and *seq4N*; the average stitching scores for video sequence pairs are shown in Figure 10.

#### 3.4.1. Effect of Homography Estimation Interval on Stitching Performance

As can be seen in Figure 10, the average stitching score for the entire sequence decreases as the homography estimation interval $T_{e}$ increases to 30. However, when $T_{e}$ is 10 in the proposed method, there are certain intervals in which the performance is lower than that of the per-frame method. This is because the time to detect representative feature points that are robust against noise in the spatio-temporal direction is not sufficient, but the average stitching score still improves due to the histogram and blob response boosted set of matched representative feature points for RANSAC. However, if the homography estimation interval $T_{e}$ increases to 30 or 40, the stitching score will saturate. For all the combinations of the homography estimation intervals and sequences, the stitching scores of the proposed method are superior to those of the per-frame method. Therefore, it is appropriate to set $T_{e}$ in a range from 20 to 40. In the previous experiments, $T_{e}$ was set to 20, the case with the lowest complexity in the range.

#### 3.4.2. Effect of Homography Estimation Interval on Processing Speed

Next, the average speed of homography estimation and stitching with respect to $T_{e}$ was evaluated. Table 3 summarizes the average processing time according to the video sequence pairs and $T_{e}$. In the per-frame method, the average stitching time up to 40 frames is calculated. The per-frame method consumes 150.4–181.9 ms for stitching one frame pair. In the proposed method, the average stitching time per frame is calculated by summing up the homography estimation times and all of the warping times of the frames in one-minute intervals of the video sequence pairs. With the proposed method, the initial latency for the homography estimation depends on the homography estimation interval. As the homography estimation interval increases, the time required for the representative feature point selection and homography estimation processes increases. However, once the homography has been estimated, the time required for video stitching is only the warping time in static camera environments. Therefore, as the length of a video sequence pair increases, the average stitching time converges to the average warping time. The proposed method requires 30.4–39.5 ms to stitch one frame pair when $T_{e}$ is set to 30.

Although the proposed method experiences some initial latency during homography estimations, it can be used for the real-time stitching of video sequence pairs. In addition, the proposed method shows less misalignment distortion of the stitched frames because it is possible to estimate a noise-resistant homography in the spatio-temporal direction. The proposed method can be more economical than the per-frame method from the viewpoint of operational cost, because it can stitch all the frames in a video sequence pair with only an initial estimation of homography in static camera environments.

In dynamic camera environments, an earlier real-time homography estimation method [16] also requires the corresponding initial homography, which mostly contributes to the accuracy levels of the homography estimation of the following video frames. The proposed method can be combined with the real-time homography estimation algorithm in the aforementioned study [16] for better dynamic homography estimations.

## 4. Conclusions

In this paper, a multi-frame based homography estimation method is proposed for video stitching in static camera environments. Homography that is robust against spatio-temporally induced noise can be estimated by intervals, using feature points extracted during a predetermined time interval. Representative feature points with the largest blob response in each quantized location bin are used for matching a pair of video sequences. After matching representative feature points from each camera, the homography for the interval is estimated by RANSAC on the matched representative feature points, with their chances of being sampled proportional to their numbers of occurrences and their blob responses in the interval.

Compared with the per-frame method, for daytime and noisy video sequences, the proposed method reduces alignment distortion in overlapped regions and improves stitching score. The increase in the average computational load by the proposed method in static camera applications is negligible if the initial homography estimated is used for an entire video sequence pair.

Since the proposed method is based on video sequence pairs captured by static cameras, it cannot be directly applied to dynamic camera environments. However, if the pose or displacement information of cameras can be used, feature points robust against noise in spatio-temporal directions can be searched for each interval and used for per-frame homography estimation for the interval.

## Figures and Tables

**Table 1:** Video sequence pairs for performance evaluation.

Name	Shooting Time	Description
*seq1*	day	tiled exterior walls of a research building
*seq2*	day	tiled exterior walls of a library building
*seq3*	day	outdoor seating area with tiled floor
*seq4*	day	corner side view of a stadium
*seq2N*	night	tiled exterior walls of a library building
*seq4N*	night	corner side view of a stadium

**Table 2:** Stitching scores for daytime video sequence pairs. $T_{e}=20$.

	Per-Frame				Proposed	
	**Frame 1**	**Frame 12**	**Frame 20**	**Frame 1–20**	**Frame 1–20**	**Improvement**
*seq1*	2.77	3.28	2.87	2.74	**2.11**	23.0%
*seq2*	4.74	3.71	3.61	4.30	**2.66**	38.1%
*seq3*	**1.78**	2.98	3.93	3.41	2.06	39.6%
*seq4*	2.95	1.08	2.58	2.03	**0.87**	57.1%

**Table 3:** Average processing time for different homography estimation interval $T_{e}$. Unit (ms).

		Per-Frame	Proposed			
			$T_{e}=$ 10	$T_{e}=$ 20	$T_{e}=$ 30	$T_{e}=$ 40
*seq1*	H. estimation time	153.3	5442.4	7325.7	9250.0	11,443.8
	warping time/frame	27.3	28.6	28.3	28.0	28.2
	stitching time/frame (1 min)	180.6	31.6	32.4	33.1	34.6
*seq2*	H. estimation time	128.5	3415.1	5268.8	6692.2	8388.7
	warping time/frame	29.0	28.9	30.2	30.8	30.3
	stitching time/frame (1 min)	157.5	30.8	33.1	34.5	34.9
*seq3*	H. estimation time	145.2	3026.6	4820.6	6304.0	8306.3
	warping time/frame	36.7	35.4	35.4	36.0	35.7
	stitching time/frame (1 min)	181.9	37.1	38.0	39.5	40.4
*seq4*	H. estimation time	122.9	2731.4	4436.7	5196.5	6436.7
	warping time/frame	27.5	27.6	27.3	27.5	29.3
	stitching time/frame (1 min)	150.4	29.1	29.7	30.4	32.9

## Notations

**Symbol**	**Description**
$T_{e}$	a homography estimation interval
$T_{n}$	the $n^{th}$ homography estimation interval from frame $\left(n-1\right)T_{e}$ to frame $nT_{e}-1$
l(p)	the location of a feature point p
R(p)	the blob response of a feature point p
|A|	the number of elements in set *A*
$p_{v}$	a representative feature point in $G_{v}^{r}$
$S_{n}$	a set of feature points in the $n^{th}$ interval $T_{n}$
$S_{n,v}$	a set of feature points in the location bin $G_{v}^{h}$ in the $n^{th}$ interval $T_{n}$
$S_{n}^{r}$	a set of representative feature points in the $n^{th}$ interval $T_{n}$
$S_{n}^{m}$	a set of matched pairs of representative feature points in the $n^{th}$ interval $T_{n}$
$\Delta_{r}$	the width of a square-quantized location bin
$\Delta_{h}$	the width of a square-quantized histogram bin
$G_{v}^{r}$	a square quantized location bin for the election of a representative feature point of
	which upper-left vertex is at v and the width is $\Delta_{r}$
$G_{v}^{h}$	a square quantized histogram bin for the histogram generation of feature locations,
	of which upper-left vertex is at v and the width is $\Delta_{h}$
$h^{n}\left(p\right)$	the number of occurrences of feature points in $G_{v}^{h}$ where p is located in the interval $T_{n}$
$v^{h}\left(p\right)$	a upper-left vertex of the quantized histogram bin where a feature point p is located
$p_{i}^{l}$	the ith left matched representative feature point
$p_{i}^{r}$	the ith right matched representative feature point
*M*	the number of pairs of matched representative feature points
H	a homography between a pair of video sequences
